# Event related potentials in children with internet addiction disorder

**DOI:** 10.1192/j.eurpsy.2021.260

**Published:** 2021-08-13

**Authors:** S. Bakhtadze, N. Khachapuridze, N. Geladze

**Affiliations:** Department Of Paediatric Neurology, Tbilisi State Medical University, Tbilisi, Georgia

**Keywords:** Latency, Internet addiction disorder, Attention, Event related potentials

## Abstract

**Introduction:**

Internet addiction disorder (IAD) is defined as one of the commonest disorder in children and adolescents affecting 40 percent of them. Although it does not cause mental disorders it is known that IAD is commonly related with attention deficit and hyperactivity disorder (ADHD). The best approach to asses attention is recording of event related potentials (ERPs) especially late response like P300. There are growing evidence regarding assessment of attention in IAD with different questionnaires but less is known about evidence received with more valid measurements like P300.

**Objectives:**

The aim of our study was to measure attention parameters in IAD subjects by using the most valid test–latency and amplitude of P300. We have examined 70 children with IAD aged 5-18 years. Children were divided into two groups: Group 1 (40 children) was matched as a study group including children with IAD and group 2 controls (30 children) without IAD and without ADHD.

**Methods:**

IAD was assessed by Young IAD scale. Children with Young scale less than 20 and with IQ less than 85 were excluded from the study. Recording of P300 was done by international protocol using oddball paradigm method. Statistical analysis was done by SSPS 26.

**Results:**

In study group increase in P300 latency was found (mean range 350-375 msc) while amplitude was normal (p <0.05). In controls both parameters were within normal range.

**Conclusions:**

IAD could be related with attention disorders causing poor attention span. This evidence is very important as they affect internet addicted children and adolescents’ social well being.

**Disclosure:**

No significant relationships.

